# Alexithymia in Amyotrophic Lateral Sclerosis and Its Neural Correlates

**DOI:** 10.3389/fneur.2018.00566

**Published:** 2018-07-24

**Authors:** Soumia Benbrika, Franck Doidy, Laurence Carluer, Audrey Mondou, Marie-Sonia Buhour, Francis Eustache, Fausto Viader, Béatrice Desgranges

**Affiliations:** Neuropsychology and Imaging of Human Memory, Caen-Normandy University, PSL Research University, EPHE, INSERM, Caen University Hospital, Caen, France

**Keywords:** amyotrophic lateral sclerosis, alexithymia, emotional processing, emotional awareness, cognitive function

## Abstract

**Introduction:** Amyotrophic lateral sclerosis (ALS) is a severe neurodegenerative disease that causes progressive and extensive motor deficits. Patients may also have cognitive impairments or alteration of emotional processing. Very few studies, however, have looked at deficits in how they experience their own feelings (alexithymia).

**Methods:** We assessed alexithymia in 28 patients with ALS using the 20-item Toronto Alexithymia Scale (TAS-20), comparing them with a control group matched for sex, age, and education level. We took into account both the total score of the TAS-20 and its three subscores corresponding to the three dimensions of alexithymia: Difficulty Identifying Feelings (DIF), Difficulty Describing Feelings (DDF), and Externally Oriented Thinking (EOT). Patients also underwent a neuropsychological assessment and anatomical magnetic resonance imaging (MRI) in order to correlate cognitive performances and gray matter volume and level of alexithymia.

**Results:** On average, ALS subjects had a significantly higher total score and DIF sub-score of the TAS-20 than controls indicating an increased alexithymia in patients. Total and DIF Scores correlated significantly and negatively to gray matter volume of the prefrontal cortex, right superior temporal pole and parahippocampal gyri. No correlations were found between scores on executive functions and those on the TAS-20.

**Conclusion:** The first stage of one's own emotional processing seems to be affected in ALS independently of executive dysfunction. This trouble seems to be underpinned by cerebral regions that are well known to be both implicated in alexithymia in healthy subjects and altered in ALS.

## Introduction

Amyotrophic lateral sclerosis (ALS) is a neurodegenerative disease of upper and lower motor neurons, with relentless progressive muscular paralysis causing severe functional disability and leading to death. Median survival is 3 years after onset of the disease and 2 years after diagnosis (1).

Approximately half of all patients with ALS have some degree of cognitive impairment, mainly affecting executive functions such as mental flexibility, attention, and verbal fluency (2). Comorbid frontotemporal dementia (FTD) is present in 5 to 15% of cases (2, 3). Patients also have impaired emotional facial expression recognition, impaired emotional responses, impaired excitability in response to emotional stimuli, and impaired judgment of emotional valence (4, 5). Affective and cognitive Theory of Mind (ToM) is also affected by the disease (6, 7), this finding having been shown to be discrepant when comparing within ALS patients, stratified by bulbar and spinal onset in some studies (8, 9), but not in others (6).

*Alexithymia* is a concept that was introduced by Nemiah and Sifneos to characterize the psychological profile of patients with psychosomatic disorders. The term literally means *lack of words for emotions*, and has been defined as “a diminution or absence of the basic human ability to experience feeling” (10). Five salient characteristics of alexithymia have been described: (a) partial or total inability to experience emotions; (b) difficulty identifying feelings; (c) difficulty describing feelings; (d) externally oriented thinking or inability to focus on one's own emotions; and (e) partial or total inability to fantasize. Larsen et al. (29) distinguished between two main forms of alexithymia. In Type-I alexithymia, the absence of both emotional experience and cognition accompanying emotion seems to be associated with psychosomatic diseases (e.g., chronic pain or gastrointestinal dysfunction). Type-II alexithymia is characterized by a selective deficit in emotional cognition, with a normal or high degree of conscious awareness of emotional arousal and sparing of emotional experience. This type of alexithymia is thought to be associated with psychiatric illness (eating disorder, anxiety, depression).

To our knowledge, alexithymia levels in patients with ALS have so far only been assessed in two studies. When Bungener et al. (11) assessed emotional experience and expressiveness in 27 patients using the Echelle d'Humeur Depressive (EHD) questionnaire, they concluded that patients have no emotional blunting or anhedonia. In their study, however, there was no control group, and the questionnaire they used mainly assesses emotional deficit (anhedonia, sadness) and loss of emotional control (impulsivity, irritability), which are encountered in depression, rather than in alexithymia. Roy-Bellina et al. (12) evaluated the alexithymia levels of 14 patients with ALS and nine healthy controls, using the 20-item Toronto Alexithymia Scale (TAS-20). TAS-20 scores were significantly higher in patients than in controls. The authors suggested that alexithymia is a defensive mechanism that protects patients against anguish and the reality of death. In this second study, groups were small and participants were not matched for sex and age. This may have biased the results, as demographic characteristics and education level are known to influence the level of alexithymia (13). Neither study assessed the neural correlates of alexithymia in ALS.

The above findings suggest that the level of alexithymia is high in some patients with ALS. As mentioned, however, alexithymia is a heterogeneous concept. We therefore set out to pinpoint the difficulties patients have processing their own emotions. Furthermore, we looked for the neural substrate in link with one's own emotional processing in ALS.

The aims of the present study were to compare patients with ALS and controls on the processing of one's own emotions in ALS patients, and to determine the relationship of any impairment with morphological and cognitive data yielded by an anatomical MRI scan and a thorough cognitive assessment.

## Materials and methods

### Participants

We recruited 28 patients with ALS for the present study. We also included 30 healthy controls. The two groups were matched for age and sex. All participants were native French speakers, were aged more than 18 years, and had more than 7 years of education. Individuals were not included in the study if they had a history of alcoholism, head trauma, or neurological or psychiatric illness. Finally, controls had to have good overall cognitive functioning, as assessed by the Mattis Dementia Rating Scale (MDRS > 130), and no patient had a severe cognitive deficit (MDRS ≥ 127) (14).

All patients were examined by an experienced neurologist (F.V. or L.C.). They met the new El Escorial criteria for probable or definite ALS (15). None of them fulfilled the criteria for a diagnosis of FTD according to the core and supporting diagnostic features of FTD detailed in the Lund-Manchester consensus statement (16) or Rascovsky et al.'s criteria (17) for a possible and/or probable behavioral variant of FTD. None had primary progressive aphasia. Genetic testing was not included in the study.

For each patient, we noted disease duration, clinical onset topography (limb vs. bulbar), the severity of ALS at the time of the study according to the ALS Functional Rating Scale revised form (ALSFRS-R), the Norris ALS Scale, which assesses the impact of bulbar involvement, and the Medical Research Council Muscle Strength Scale (MRC scale). Patients were able to speak and/or write intelligibly, had a forced vital capacity above 50% of the predicted value, and no clinical evidence of nocturnal hypoventilation. None of the patients had any additional severe or chronic illness, MRI contraindications, or communication difficulties severe enough to compromise the administration of cognitive tests. They gave their written informed consent, and the study was approved by the regional independent ethics committee.

### Alexithymia level measures

The level of alexithymia was gauged with the validated French version of the 20-items Toronto Alexithymia Scale (TAS-20) (18), a reliable self-report scale and the most widely used measure of alexithymia (19, 20). Each of the 20 items is rated on a 5-point scale ranging from 1 (*strongly disagree*) to 5 (*strongly agree*). The TAS-20 assesses three dimensions of alexithymia, by means of three specific subscales: Difficulty Identifying Feelings (DIF) is measured by summing responses to Items 1-3-6-11-9-13-14, Difficulty Describing Feelings (DDF) by summing responses to Items 2-4-7-12-17 and Externally Oriented Thinking (EOT) by summing responses to Items 5-8-10-15-16-18-19-20 (19). We thus obtained a TAS-20 total score and one additional subscore for each dimension.

### Cognitive and behavioral functioning assessment

Global cognitive functioning was gauged with the widely used MDRS, which provides a sensitive measure of the degree of frontal-subcortical impairment (21). Both patients and controls underwent an additional neuropsychological assessment that included the second part of the French version of the Hayling Sentence Completion Test (HSCT) to evaluate the ability to inhibit a dominant response (number of correct responses) (22); the Letter-Number Sequencing task (LN sequencing) to measure the ability to manipulate items in working memory (rearrangement and transformation of representations for goal-directed behavior) (23); and the Trail Making Test (TMT) and letter verbal fluency task (VF) to evaluate set-shifting abilities (24). The TMT comes in two parts: the time taken to process Part A yields a measurement of processing speed, while that taken to process Part B minus the processing time for Part A (TMT B-A) measures the ability to flexibly shift course during an activity. The verbal fluency performances of patients with a speech impairment were expressed by an index based on the number of words produced in the VF task and the time it took them to read out the words in a subsequent reading task (25). To evaluate if patients were cognitively impaired, we calculated the *Z*-scores for each patient using means and standard deviations of the control group with pathological threshold defined at *Z* = ±2.

To assess behavioral changes induced by the disease, we have proposed the French version of the Neuro-Psychiatric-Inventory Questionnaire (NPI-Q) to relatives. This questionnaire is composed of 12 items corresponding to 12 symptoms: apathy, delirium, hallucination, etc. Caregivers had to note if each symptom is present or absent and if present they have to evaluate its severity (from 1 to 3) and its impact on themselves (from 1 to 5) (26). We thus obtained three scores for each patient: number of symptoms (/12), severity of symptoms (/36), and caregiver distress (/60).

### MRI data acquisition

For each patient, a high-resolution T1-weighted anatomical image was acquired on an Achieva 3T scanner (Philips, Eindhoven, The Netherlands) using a three-dimensional fast field echo sequence (sagittal; repetition time = 20 ms, echo time = 4.6 ms, flip angle = 20°, 180 slices with no gap, slice thickness = 1 mm, field of view = 256 × 256 mm^2^, in-plane resolution = 1 × 1 mm^2^). The MRI examination was carried out within 24 h of the clinical testing.

The MRI data were segmented, spatially normalized to Montreal Neurological Institute (MNI) space (voxel size = 1 mm^3^), modulated to correct for nonlinear warping effects, and smoothed with a 10-mm full width at half maximum Gaussian kernel using the VBM5 toolbox implemented in SPM5 software (www.fil.ion.ucl.ac.uk). Images were masked to exclude non-gray matter (GM) voxels from the analyses.

### Statistical analysis

Statistical analyses of demographic characteristics, alexithymia level and cognitive data were performed using STATISTICA 10.0 (StatSoft, Tulsa, OK, USA). The threshold of significance was set at *p* = 0.05. As we expected a higher level of alexithymia as already shown by Roy-Bellina et al. (12) and lower performances to cognitive tests in patients versus controls, we performed between-group comparisons using one-tailed thresholds. Regarding correlations, as we had no a priori hypothesis, analyses were done using two-tailed thresholds.

As some variables were not normally distributed, non-parametric tests were used. U Mann-Whitney test was employed to compare the patients and healthy controls on age, years of education, TAS-20 scores, MDRS score, TMT B-A reaction time difference, verbal fluency score and index, HSCT score and LN sequencing score. A chi-square test allowed us to compare sex ratio and proportion of non-alexithymic versus alexithymic participants across the two groups. In the patient group, correlations between TAS-20 scores and cognitive performances were calculated with Spearman's correlation coefficient.

Finally, correlations between impaired TAS-20 total and DIF scores and whole-brain GM volume were calculated using the multiple regression routine of Statistical Parametric Mapping (SPM 5; Wellcome Trust Center for Neurology, London, UK) across the 28 patients. In line with our main objectives, we focused on the negative correlations (we expected a higher level of alexithymia to be correlated with lower GM volume). Given the deleterious effect of age on GM volume and the potential effect of educational level on alexithymia, the patients' ages and educational levels were entered as confounding variables. We used a statistical threshold of *p* = 0.005 (uncorrected for multiple tests) for the voxels and a cut-off of k (corresponding to the number of voxels in a particular cluster) >100. Anatomical localization was based on Talairach's atlas and the Anatomical Automatic Labeling atlas [AAL; (27)]. We used the MNI template of SPM 5.

## Results

### Demographic, clinical, behavioral, and neuropsychological characteristics of the two groups

The demographic, clinical, and neuropsychological characteristics of the two groups are shown in Table 1. Patients and controls were matched for sex, ${\chi \;}_{\left(1\right)}^{2}$ = 0, *p* = 0.99, age, = 0.09, but not for education level, *p* = 0.02. Regarding global cognitive and executive functioning, patients scored lower than controls on the MDRS, *p* < 0.001, HSCT, *p* < 0.001, TMT B-A, *p* = 0.01, LN sequencing task, *p* < 0.01, letter verbal fluency scores, *p* < 0.001, and index, *p* < 0.001). All patients were impaired in at least one cognitive test but only 57 % of them were defined as “cognitively impaired,” meaning that they were deficient in at least two tests. Scores obtained at the NPI-Q are reported in Table 1.

**Table 1:** Participants' demographic and clinical characteristics and cognitive performances.

	**Patients**	**Range**	**Controls**	**Range**
Sex (M/F)	15/13		16/14	
Age (years)	61.28 (± 11.17)	43–81	57.30 (± 9.72)	45–75
Education (years)^*^	9.68 (± 2.59)	7–17	11.03 (± 2.57)	7–16
Onset (bulbar/limb)	5/23			
Disease duration (months since clinical onset)	17.89 (± 6.37)	5–44	\	\
ALSFRS-R score/48	37.57 (± 5.89;	27–48	\	\
Norris score/39	35.10 (± 5.65)	16–39	\	\
MRC score/120	99.78 (± 15.29)	63–120	\	\
MDRS^**^	136.39 (± 5.34)	127–144	142.40 (± 2.13)	130–144
HSCT correct responses^**^	5.89 (± 3.02)	0–12	9.00 (± 3.14)	4–14
TMT B–A (s)^*^	73.25 (± 86.59)	8–371	34.55 (± 30.90)	3–121
Letter verbal fluency scores^**^	15.11 (± 4.43)	8–25	24.59 (± 5.75)	13–38
Letter verbal fluency index ^**^	6.89 (± 2.27)	5–13	4.35 (± 1.24)	3–8
LN sequencing score ^**^	8.46 (± 3.63)	3–17	10.48 (± 2.06)	8–16
NPI-Q number of symptoms	2.08 (± 1.55)	0–4	\	\
NPI-Q severity of symptoms	3.91 (± 3.22)	0–12	\	\
NPI-Q caregivers distress	5.20 (± 4.25)	0–15	\	\

*Values are means (^+/−^ SD). ALSFRS-R, Amyotrophic Lateral Sclerosis Functional Rating Scale-revised form; MRC, Muscle Strength Scale; MDRS, Mattis Dementia Rating Scale; HSCT, Hayling Sentence Completion Test; TMT, Trail Making Test. NPI-R, Neuro-Psychiatric Inventory Questionnaire*.

* *p < 0.05*.

** p < 0.001. Disease duration was the period between clinical onset and the date of patient inclusion in the study

### Alexithymia level

Regarding alexithymia level, and as indicated in Table 2, the mean TAS-20 total and DIF subscores were significantly higher in patients than controls. By contrast, even though patients scored higher than controls on the other two subscores (DDF and EOT), the differences were not significant. A total of 53.6% of patients were alexithymic (TAS-20 total score >51), compared with just 23.3% of controls, $\chi_{\left(1\right)}^{2}$ = 5.62, *p* = 0.02.

**Table 2:** Scores on the 20-item Toronto Alexithymia Scale and proportion of alexithymic versus nonalexithymic participants.

	**Patients**	**Controls**	***P***
TAS-20 Total	49.36 (±13.38)	42.80 (± 10.06)	0.03^*^
TAS-20 DIF (/35)	15.78 (± 6.05)	12.43 (± 5.03)	0.01^*^
TAS-20 DDF (/25)	15.21 (± 4.93)	13.73 (± 5.07)	0.12
TAS-20 EOT (/40)	18.32 (± 4.86)	16.63 (± 3.46)	0.08
A/NA	15/13	7/23	0.02^*^

*TAS-20, Toronto Alexithymia Scale; DIF, Difficulty Identifying Feelings; DDF, Difficulty Describing Feelings; EOT, Externally Oriented Thinking; A, alexithymic; NA, nonalexithymic*.

* *p < 0.05*.

In the patient group, TAS-20 scores correlated with age (*r* = 0.41, *p* = 0.03), but not with education level, clinical data (ALSFRS-R score, Norris score and MRC score), MDRS score, behavioral status (scores of the NPI-Q) or executive function performances (HSCT, TMT B-A, LN sequencing score and letter verbal fluency score and index).

### Correlations between alexithymia level and GM volume

**Figure 1:**
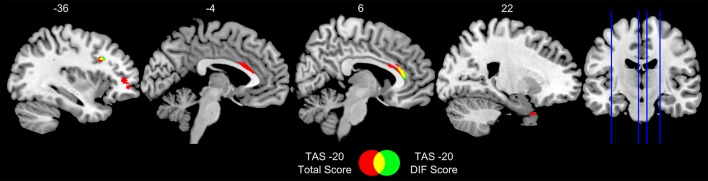
Sagittal views of regions in which gray-matter volume correlated significantly and negatively with TAS-20 total and DIF scores in 28 patients with ALS. TAS-20 = 20-item Toronto Alexithymia Scale; DIF, Difficulty Identifying Feelings. Results are displayed at *p* < 0.005 uncorrected and k > 100 mm3. Numbers in the figure indicate x coordinates in MNI space.

The peaks of the significant negative correlations between the TAS-20 total or DIF scores and GM volume, with age and educational level as confounding variables for 28 patients, are given in Tables 3, 4. TAS-20 total scores correlated negatively and significantly with GM volume in the right and left anterior cingulate cortex (ACC), left inferior frontal gyrus (triangular and opercular parts), left middle frontal gyrus, and right superior temporal gyrus (see Figure 1). TAS-20 DIF scores had significant negative correlations with GM volume in the right ACC, left inferior frontal gyrus (triangular and opercular parts), and middle frontal gyrus (see Figure 1).

**Table 3:** Mean peaks and regions of significant negative correlations between TAS-20 total score and gray-matter volume (*p* ≤ 0.005).

**MNI coordinates**			**Labels**	**K**	**Z (voxel label)**
**x**	**y**	**z**			
−33	15	30	Frontal_Inf_Oper_L	203	3.83
			Frontal_Inf_Tri_L		3.68
			Frontal_Mid_L		
6	29	19	Cingulum_Ant_R	435	3.68
−36	45	4	Frontal_Mid_L	232	3.60
			Frontal_Inf_Tri_L		
			Frontal_Mid_Orb_L		
−4	18	24	Cingulum_Ant_L	143	3.24
22	13	−33	Temporal_Pole_Sup_R	111	3.23
			ParaHippocampal_R		
			Temporal_Pole_Mid_R		

*Labels were obtained using the AAL toolbox (see Materials and methods). K, cluster size; L, left; R, right; Oper, opercular; Tri, triangular; Orb, orbital; Inf, inferior; Ant, anterior; Sup, superior; Mid, middle*.

**Table 4:** Mean peaks and regions of significant negative correlations between TAS-20 DIF score and gray-matter volume (*p* ≤ 0.005).

**MNI coordinates**			**Labels**	**K**	**Z (voxel label)**
**x**	**Y**	**z**			
8	37	10	Cingulum_Ant_R	375	4.04
−32	14	30	Frontal_Inf_Oper_L	147	3.67
			Frontal_Mid_L		
			Frontal_Inf_Tri_L		

*Labels were obtained using the AAL toolbox (see Materials and methods). K, cluster size, L, left, R, right, Oper, opercular, Tri, triangular, Orb, orbital, Inf, inferior, Ant, anterior, Sup, superior, Mid, middle*.

## Discussion

### Alexithymia in ALS

Our results confirm that both the experience and the expression of one's own emotions are altered in a high proportion of patients with ALS. These patients have significant difficulty identifying their feelings compared with healthy individuals. More than half of patients are alexithymic, whereas the prevalence of alexithymia in the general population ranges from 2 to 19%, depending on age, sex and educational level (13, 28). The prevalence of alexithymia in our control group was 23%, which approximates to the highest rates in general population, probably because of the predominance of men and the advanced age of the group.

The TAS-20 scale that was not designed to specifically identify Type-I or Type-II alexithymia, tends to emphasize its cognitive aspects (identifying, verbalizing, and analyzing) and to underestimate its emotional component, represented by emotionalizing and fantasizing (29). However, if we assume that the DIF subscore primarily reflects emotional experience (including first the perception of physical sensations and then an awareness of their significance) whereas the other two subscores reflect emotional mentalizing, then the main difficulty of patients with ALS would appear to lie in the experiencing of emotion, which corresponds to Type-I alexithymia. Interestingly, Larsen et al. (29) suggested that a substantially reduced level of emotionalizing protects individuals from emotional and psychiatric problems. It is a matter of fact that fewer than 10% of patients with ALS experience depression (30, 31), which could partly be due to their impaired emotional experience.

The clinical importance of a high level of alexithymia in ALS remains to be specified. Type I alexithymia could induce psychosomatic manifestations, which could in turn skew the ALS patients' physical condition and make their medical management more difficult if psychological care is not proposed. High levels of alexithymia have been found to be correlated to less capacity of empathy [see e.g., (32)]. As shown by Lockwood et al. (33) a low capacity of empathy is associated to apathy, a behavior that increases the burden of ALS caregivers (34). Thus, a lack of empathy potentially induced by the high level of alexithymia in some patients could increase the burden of caregivers. Identifying and treating this psychological condition in patients is crucial to prevent suffering of relatives.

Alexithymia has been found to impair quality of life (QoL) in the general population (35), and in several diseases as well, but not in ALS, to the best of our knowledge. Further studies are needed to shed light on the clinical consequences of high level of alexithymia in ALS.

### Alexithymia and cognitive and emotional deficits in ALS

Our group of patients is in line with the generally accepted finding that in ALS about half of the subjects have “normal cognition” and half have some sort of “cognitive impairment” (36). Even if there are no normative data to interpret the NPI-Q, patients have very little behavioral disorders compared to what is reported in pathology like frontotemporal dementia (37). Behavioral impairment occurs in around 30% of ALS patients (8, 38) it is usually associated to cognitive impairment but may be isolated in 6% of cases (36). Behavioral changes mostly include disinhibition and apathy. The most encountered subtype of apathy in ALS is the lack of initiation, a lack of motivation to self generation of thoughts (39). This subtype is in link with poorer verbal fluency performances, which suggests that apathy in ALS is underpinned by the medial prefrontal cortex (39).

We found no correlations between our patients' set-shifting abilities, working memory and ability to inhibit responses, and their alexithymia level. A high level of alexithymia has been found to be associated with impairment of executive functions as assessed using the TMT and verbal fluency tasks (40–42). But this association concerned mainly DDF, sometimes EOT or Total alexithymia (40–42). Furthermore, Paradiso et al. (43) reported a significant association in healthy volunteers between the total alexithymia score and scores on the Controlled Oral Word Association test. These results suggest that alexithymia may be linked not only to impaired executive functions but also possibly to poor linguistic abilities. Thus, only the cognitive aspects of alexithymia (DDF and EOT dimensions) are linked to executive function or language abilities. The absence of a correlation between executive function and level of affective alexithymia (DIF dimension) could be explained by the fact that the first stage of emotional processing does not involve either executive functions or language abilities. The impaired processing of emotions in the self could thus be *specific*, and partly independent of executive dysfunction in ALS.

Patients with ALS are widely acknowledged to have deficits in recognition of other people's emotions, and in both cognitive and affective ToM (4, 44). In some studies, executive functions have been found to be correlated with affective and cognitive ToM (44, 45) but alteration of ToM in ALS have also been shown to be partially independent of executive function (44, 46). van der Hulst et al. (46) showed that deficits in ToM are associated to poorer self-awareness. Global emotional and social dysfunctions are potentially underpinned by one's own emotional processing deficit. Indeed, in order to understand the mental states of others, individuals must activate representations of those states (i.e., simulate how others are feeling) within themselves (47). Patients' social cognition impairment may therefore stem from the difficulty they have representing their own emotions (because of a lack of emotional experience), insofar as this makes it harder for them to mentalize what others are feeling or thinking.

### Alexithymia and its neural correlates in ALS

#### Involvement of the anterior cingulate and prefrontal cortices in the processing of emotions in the self

This is the first study to have assessed the correlation between GM volume and alexithymia level in ALS. We found significant negative relationships between the TAS-20 total score and DIF subscore and the GM volume of the prefrontal cortex (inferior and middle frontal gyrus) and ACC. In other words, patients with a high level of alexithymia had reduced GM volume in these areas. These results are consistent with observations in healthy individuals, as several neuroimaging studies have reported decreased GM volume in the ACC and medial prefrontal cortex, in healthy individuals with a high level of alexithymia (48–51).

Reductions in GM density in ALS have been observed in the frontal lobe and, more specifically, in the inferior (52, 53), middle and superior frontal gyri (53) and ACC (54, 55). These areas partially overlap with those thought to be involved in the processing of one's own emotions, which is consistent with the emotional processing alterations found in a number of patients with ALS. Lane et al. (56) suggested that alexithymia could be an “emotional equivalent” of blindsight, which is when an individual claims to be blind, but responds with high accuracy in visual tracking and other selective visual tasks. Extra-striate pathways allow accurate responses to some stimuli that the individual denies seeing, owing to a partial lesion in area V1 of the primary visual cortex that prevents conscious visual perception. Mirroring this description, alexithymia could be a deficit in emotional awareness caused by an impaired connection between subcortical emotion-generating mechanisms and cortical mechanisms, including the ACC, which is involved in the explicit processing of emotion. Lane et al. also found increased rostral ACC and ventromedial prefrontal cortex (VMPFC) activation when they asked healthy individuals to selectively attend to their emotional responses to various images they were shown, and to classify these emotional responses as pleasant, unpleasant or neutral (57). These authors suggested that the ACC and VMPFC play a specific part in generating representations of one's subjective emotional responses and regulating subcortical responses. Several more recent studies in the general population have shown that the rostral ACC and surrounding VMPFC regions are involved in recognizing the processing of one's emotions, independently of how these emotions are expressed.

#### Dimensions of alexithymia and their neural correlates

In our study, the total scores of the TAS-20 were correlated with three clusters (ACC, inferior and middle frontal gyri, and temporal areas), whereas for the DIF subscore, only the ACC (the biggest cluster) and the inferior and middle frontal gyri were found to be correlated. There have been a few studies of the neural correlates of the various dimensions of alexithymia. Demers et al. (58) found a correlation between GM thickness in the dorsal ACC and DIF scores in children with posttraumatic stress disorder. On a large sample of healthy subjects, Grabe et al. (51), looked for correlations between the gray matter volume and alexithymia, distinguishing the three dimensions. For the total score of the TAS-20, a significant negative correlation was found with the bilateral ACC and the right middle cingulate cortex whereas for the DIF score, bilateral ACC, bilateral middle cingulate cortex, inferior temporal and fusiform gyri were the main clusters of negative significant correlations. It is not clear if each dimension of alexithymia implies a specific network, but the cingulate cortex seems to have a transversal role in one's own emotional processing. Developing the initial blindsight neural model of alexithymia proposed by Lane et al. (56), with the aid of Marr's hierarchical model of vision, Prinz (59, 60) [cited in Smith and Lane (61)] proposed a hierarchical model of conscious and unconscious emotional self-perception with a three-stage algorithm. Stage 1 (*discrete body features*) corresponds to the perception of changes in the activity of discrete parts of the body (e.g., changes in heart rate, breathing, temperature, muscle tension). This bodily perception is mediated by the somatosensory cortex. In Stage 2 (*whole body patterns*), discrete Stage 1 representations are integrated in order to detect/represent coherent whole-body patterns. The anterior and middle insula and dorsal ACC may play a crucial role in this stage (61). Stage 3 (*emotion concept*) involves mentalizing the integrative whole-body perception and giving meaning to what is perceived and felt. Likely candidates for Stage 3 emotion processing are the rostral ACC and adjacent regions of the medial prefrontal cortex (MPFC). Different stages in this emotion processing presumably correspond to different alexithymia dimensions, with DIF corresponding to Stage 1 or 2 of Prinz's model insofar as this dimension reflects a perceptual process of relatively low cognitive level, and DDF and EOT to Stage 3. Our results support the idea that the ACC has an integrative function for perceived sensations, while the inferior and middle frontal gyri are involved in mentalizing emotions.

#### The temporal lobe and the processing of one's own emotions

Cortical damage in ALS may involve both temporal and limbic areas (52, 53). In our study, a deficit in the processing of one's emotions was associated with reduced GM volume in the right superior and middle temporal pole. Healthy individuals' alexithymia scores are reported in the literature to be correlated with the GM volume of both the superior temporal pole and the parahippocampal region (49, 50). Parts of the temporal lobe are known to be involved in facial emotion recognition and social cognition (62). The superior part is involved in lexical and semantic retrieval, while the anterior part seems to be an associative structure that links representations of meaning (63). Cerebral lesions or neurodegenerative disease affecting the temporal pole are known to cause behavioral, emotional and social impairments, e.g., disinhibition, hypersexuality, eating disorder, emotional blunting, apathy and inadequate social responses (64). Neuroimaging studies have shown that the temporal pole is involved in tasks requiring to think about thoughts and emotions of others (64, 65). The right temporal pole is more specifically implicated in emotional processing, social behavior and personal and episodic memories whereas the left temporal pole is more engaged in semantic processing (64, 66). Pehrs et al. (67) have suggested that the temporal pole integrates information from different modalities and has a modulating effect on perceptual areas. This could explain its implication in alexithymia and, more specifically, in the other dimensions besides DIF.

Our study has some limitations. The first one is that as the patients are only mildly physically affected and may thus not be representative of an ALS standard population, generalization of the results must be done with caution. Another limitation is that our threshold of significance for neural correlates has been set at *p* < 0.005 (uncorrected for multiple tests). This threshold is not very robust but our results are consistent with the few relevant data in the literature, which gives credit to our study. The fact that the TAS-20 scale only captures three of the five previously described dimensions of alexithymia may be a methodological limit of the present study. Finally, we focused on executive functions because these are the most impaired in ALS patients and thus implications of other cognitive domains in alexithymia were not studied. Further studies are needed, first to validate our findings and then to better assess the clinical implications of alexithymia in ALS, in terms of QoL of both patients and caregivers for example. It would also be interesting to stratify the alexithymia data along clinical and genetic subtypes of the disease.

## Conclusion

To our knowledge, this is the first study assessing alexithymia in patients with ALS compared with a matched control group, specifically focusing on the various dimensions of alexithymia. Our results add to the accumulating evidence that a number of central nervous functions are impaired in ALS besides motor control, including the processing of emotions in the self. These emotional disabilities could explain patients' paradoxical psychological reactions. In ALS, emotional self-perception involves brain regions that are known to contribute to emotion processing in healthy individuals. Even if the nature of emotional neurological circuits has been well established, the precise role of specific neurological structures remains to be delineated. ALS could be a good model for improving current understanding of the emotional brain.

## Data availability statements

The raw data supporting the conclusions of this manuscript will be made available by the authors, without undue reservation, to any qualified researcher.

## Ethics statement

All subjects gave their written informed consent, and the study was approved by the regional independent ethics committee.

## Author contributions

SB was actively involved in this study from design to drafting. She conduced all the statistical analyses and played a central role in interpreting the results and writing the article. FD and M-SB greatly contributed to the neuroimaging part of the study. AM and LC participated in the acquisition of clinical and cognitive data, especially in the careful screening of our cohort. FD, LC, and FE provided their critical revision of the manuscript. BD and FV supervised and coordinated the teamwork from start to finish. Their Knowledge and expertise in neuropsychology and ALS pathology were crucial for the design, analyses and interpretation of the result of the project. They were also particularly involved in the revising of the manuscript. All authors read and improved the final manuscript.

### Conflict of interest statement

The authors declare that the research was conducted in the absence of any commercial or financial relationships that could be construed as a potential conflict of interest.
